# Severe Bronchomalacia in a 65-Year-Old Man Treated With Stent Insertion Instead of Surgical Bronchoplasty

**DOI:** 10.1155/DTE.1.227

**Published:** 1995

**Authors:** Tom G. Sutedja, Jan C. de Graaf, Franz M. Schramel, Klaas W. Van Kralingen, Pieter E. Postmus

**Affiliations:** 1 Departments of Pulmonary Medicine ziekenhuis de Heel Zaandam and the Free University Hospital Amsterdam The Netherlands; 2 FCCP Department of Pulmonary Medicine Free University Hospital P.O. Box 7057 Amsterdam 1000 MB The Netherlands

## Abstract

Bronchomalacia in adults is rare. A case is reported here of a 65-year-old man with severe cough and mucostasis, caused by a benign bronchomalacia of the ventral wall of the left main bronchus. This was treated successfully with the insertion of a silicone Dumon stent as an alternative to surgical bronchoplasty.


*Diagnostic and Therapeutic Endoscopy*, 1995, Vol. 1, pp. 227–228
Reprints available directly from the publisher
Photocopying permitted by license only

© 1995 Harwood Academic Publishers GmbH
Printed in Singapore

### Severe Bronchomalacia in a 65-Year-Old Man Treated with Stent Insertion Instead of Surgical Bronchoplasty

TOM G. SUTEDJA, JAN C. DE GRAAF, FRANZ M. SCHRAMEL, KLAAS W. VAN KRALINGEN, and PIETER E. POSTMUS

*From the Departments of Pulmonary Medicine ziekenhuis de Heel, Zaandam and the Free University Hospital Amsterdam, the Netherlands*

*(Received April 22, 1994; in final form October 24, 1994)*

Bronchomalacia in adults is rare. A case is reported here of a 65-year-old man with severe cough and mucostasis, caused by a benign bronchomalacia of the ventral wall of the left main bronchus. This was treated successfully with the insertion of a silicone Dumon stent as an alternative to surgical bronchoplasty.

**KEY WORDS:** bronchomalacia, stent

### INTRODUCTION

Large airway obstruction leads to symptoms such as cough, mucus retention, and obstructive pneumonia. Currently available endoscopic methods may provide palliation in cases with airway obstruction caused by intraluminal tumor, but stent insertion is the only choice for malignant extraluminal tumors. Benign tracheobronchial obstruction is rare, and until recently surgery was considered the only treatment alternative. We report our experience in a patient who had a severe bronchomalacia caused by the prolapse of the ventral wall of the left main bronchus. A stent insertion was attempted as a less invasive treatment alternative for surgical bronchoplasty.

### CASE REPORT

A 65-year-old man was referred to our hospital because of left main bronchus obstruction. He was known to have COPD for several years and has been seen repeatedly by the pulmonologist because of persistent cough. Bronchoscopy had been performed 1 year before, and, showed a slight thickening of the ventral wall of the left main bronchus. One month before referral, he was admitted again because of progressive cough and chest pain. On physical examination, the temperature was normal and expiratory wheezing was heard over the left hemithorax. On bronchoscopy, a tumor in the ventral wall of the left main bronchus was found, causing an almost complete occlusion. Biopsy and cytology brush and washing were negative for malignant cells. The patient was referred to our hospital.

History did not reveal any childhood diseases, and his COPD seemed to be stable with the use of budesonide and salbutamol inhalers. He complained of gradual worsening of cough and copious sputum production in the last 5-year period. Vital capacity was normal, 3.6 1; forced expiratory volume in 1 second was 2.4 1 (predicted, 2.7 1). The flow volume curve showed slight expiratory disturbance. High-resolution CT scan revealed a slight thickening of the ventral wall of the left main bronchus without signs of infiltrative growth into the peribronchial tissue, and there were no enlarged lymph nodes. However, on bronchoscopy, a severe prolapse of the ventral wall of the left main bronchus was seen, causing a complete closure of the main bronchus during expiration and *approximately* 60% stenosis during inspiration. Distal to the stenosis, copious and tenacious mucus was present. The longitudinal axis of the narrowed part was less than 1 cm. The mucosa appeared normal (see Fig. 1). Biopsies and transbronchial cytologic examinations were normal. A Dumon stent of 2

Address for correspondence: G. Sutedja, MD, FCCP, Department of Pulmonary Medicine, Free University Hospital, P.O. Box 7057, 1000 MB Amsterdam, The Netherlands.

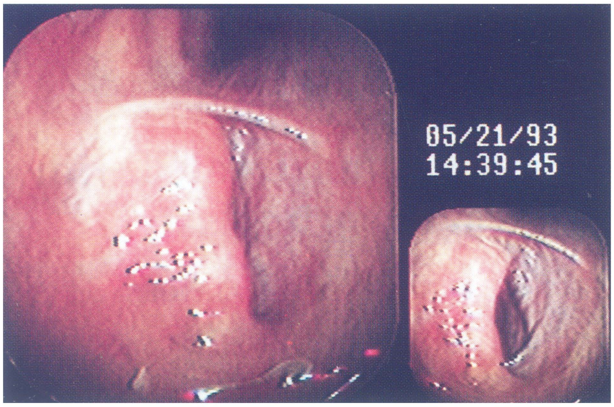

**Figure 1** Severe dynamic stenosis of the left main bronchus of unknown cause. The picture taken by videobronchoscopy during expiration showed complete obstruction of the left main bronchial lumen. During maximal inspiration, there was still approximately 60% narrowing of the bronchial lumen.

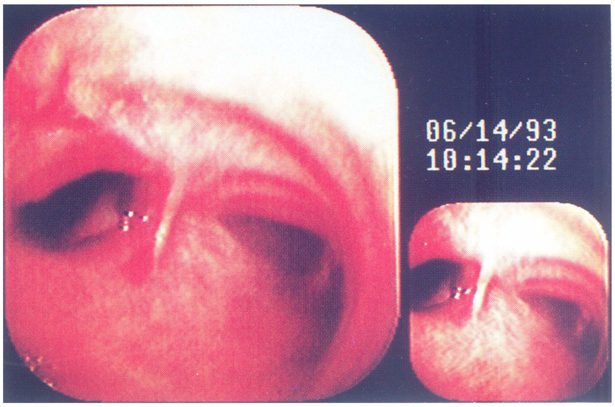

**Figure 2** Overview of the distal tracha and bronchial bifurcation after the insertion of a silicone stent. Pictures show stability of the left main bronchus during inspiration and expiration (small and large square, respectively).

cm × 16 mm (silicone Endoxane[R]) was placed under general anesthesia, and no complication has occurred. Repeated CT scan and bronchoscopy have been negative for malignancy, and follow-up has been 10 months and uneventfull.

### COMMENT

In adulthood, bronchomalacia is rare (1,3). Our patient suffered from symptoms caused by a severe prolapse of the ventral wall of the left main bronchus. Instability of the pars membranacea was reported to be the cause of bronchomalacia in patients with severe COPD and/or chronic cough (5). In our patient, the reason for this peculiar phenomenon is unknown. Malignancy is unlikely. It is possible that there is an inborn structural weakness, which became more severe in due course of his chronic cough.

Silicone stents have been used for palliation in malignant airways obstruction. Stent has also been used for benign cases such as anastomosis disruption after bronchoplastic surgery or stenosis after lung transplantation (4). This patient had a rare bronchomalacia with severe dynamic stenosis of unknown cause. A surgical approach would have make a sleeve resection necessary. Recently the use of metal expandable stents has been reported as a less invasive treatment alternative for bronchoplastic surgery (2). However, metal stents become gradually imbedded in bronchial tissue and may lead to fibrotic changes, which may compromise future surgical attempt. Our choice of the silicone stent was based on the fact that it is easier to remove in case a surgical bronchoplasty becomes necessary. The stent insertion seems to be an effective treatment alternative for surgical resection and a wait-and-see approach seems justifiable, because follow-up has been uneventful.

### REFERENCES

1. Baxter JD, Dunbar JS. Tracheomalacia. Ann Otol 1963;72:1012.
2. Von Berger H, Gärtner Ch, Kohz P, Stäbler A, Dienemann H, Wilmes E. Implantation elastischer metallendoprothesen bei trachealstenosen und tracheomalazie. Fortschr Röntggenstr 1993;159:43–49.
3. DiRienzo S. Functional bronchial stenosis. Surgery 1950;27:853.
4. Dumon JF. A dedicated tracheobronchial stent. Chest 1990;97:328–332.
5. Jokinen K, Palva T, Sutinen S, et al. Acquired trachebronchomalacia. Ann Clin Res 1977;9:52–57.

